# Commentary: A comparative randomized clinical trial evaluating the efficacy and safety of tacrolimus *versus* hydrocortisone as a topical treatment of atopic dermatitis in children

**DOI:** 10.3389/fphar.2024.1372259

**Published:** 2024-02-29

**Authors:** Jane Harvey, Beth Stuart, Hywel C. Williams

**Affiliations:** ^1^ Centre of Evidence Based Dermatology, School of Medicine, University of Nottingham, Nottingham, United Kingdom; ^2^ Wolfson Institute of Population Health, Queen Mary University of London, London, United Kingdom

**Keywords:** hydrocortisone, tacrolimus, randomized controlled trial, atopic dermatitis, commentary

## Introduction

We commend Mohamed *et al* for undertaking an independent randomized controlled clinical trial comparing the effects of 0.03% topical tacrolimus ointment against 1% hydrocortisone cream in Egyptian children with atopic dermatitis (AD). Although vehicle-controlled studies can establish whether a treatment works, active comparators are needed to provide doctors and patients guidance on how new treatments compare with established treatments (Wilkes et al., 2016; Williams, 2021). The trial was prospectively registered and showed that the short-term effectiveness of 0.03% topical tacrolimus is very similar to that of a very weak topical corticosteroid (Bowie et al., 2022) in children with AD. The trial also provided some useful data on adverse events. Some critically important aspects of the trial require further clarification before they can be accepted as credible.

## Trial duration

The prospectively registered trial protocol clearly stated that the trial duration was 4 months and not 3 weeks as stated in the final published article. The final submitted online version of the trial (which has since been removed from the *Frontiers* website) also stated that the trial duration was 4 months. There is no mention of a 3-week treatment period in the registered protocol or the nascent submitted online version, and there is no mention of the *American Academy of Pediatrics* statement (references in the article) that was introduced as justification for the 3-week period in the final version. Changing the duration of a trial or timing of a primary outcome assessment is a major trial amendment that needs to be documented carefully in an audit trial in the trial registration record along with evidence of oversight committee and ethics approval. Something strange has happened in the editorial process between submission and the final version, which requires explanation.

## Choice of primary outcomes

While clinical effectiveness using a modified Eczema Area and Severity Index (EASI) score is a reasonable primary outcome, we question the clinical relevance of measuring the three cytokines, IL-10, IL-17, and IL-23. No hypothesis is stated, and it is unclear how measuring only three of the many cytokines mentioned in the *Introduction* section is likely to improve management of atopic dermatitis. The authors conclude that “0.03% tacrolimus ointment is more beneficial than hydrocortisone cream in managing children with atopic dermatitis in terms of lowering the inflammatory markers,” yet children do not attend hospital clinics complaining of raised inflammatory markers.

## Unclear randomization process

Although the study is described as a “simply randomized clinical trial,” we note that each treatment arm has ended up with exactly 100 patients each. The probability of ending up with exactly 100 patients in each arm from simple randomization is very low indeed (Altman and Doré, 1990), raising concerns over the integrity of the randomization process and subsequent concealment of allocation.

## Sample size rationale

Although the power and significance level for the “serum dermatitis severity scale” are mentioned, the magnitude of the change being sought (delta) is not mentioned. Instead, a reference is provided for a previous study (Breuer et al., 2005), which, on reading further, also does not provide any rationale for the study sample size.

## Lack of blinding

The study is described as “double-blind,” which presumably implies that both patients and assessors were blinded to the intervention (Schulz and Grimes, 2002). Given that the 0.03% tacrolimus used in the study was an ointment and the 1% hydrocortisone was a cream, blinding was clearly not possible despite going to the expense of ensuring that the tubes were identical in size and appearance and labeling. Blinding is likely to be further comprised by the fact that 60% of those allocated to tacrolimus developed skin burning compared with 12% in the hydrocortisone group.

## Implausibility of skin atrophy data

The authors report that the rate of skin thinning in the hydrocortisone arm was 8/100 participants. That eight children should develop significant cutaneous atrophy only after 3 weeks of a very weak topical corticosteroid sounds implausible and is not supported by other large studies (Lax et al., 2022). The method of how skin atrophy was assessed is not described, e.g., whether images were taken at baseline and after 3 weeks. It is possible that such an implausible result was due to lack of blinding and strong prior beliefs against the use of topical corticosteroids by the study assessors. We would welcome any details of the recorded atrophy events, particularly whether participants had to stop treatment and whether the atrophy resolved as a result.

## Ethical concerns

We note that approximately 50% of the study children had severe atopic dermatitis, and approximately 90% had moderate or severe disease. Because 1% hydrocortisone is considered a very weak anti-inflammatory topical corticosteroid, we question the ethics of subjecting children with severe AD to such weak topical treatments for 3 weeks. Given the low potency of the treatments being tested, perhaps the trial should have been designed to restrict participation to only those with mild AD.

## Discussion

Undertaking randomized controlled trials to acceptable standards is not easy, and we appreciate the efforts of the authors in conducting this study. There are hints throughout the article that the authors clearly favored topical tacrolimus, yet the clinical effectiveness data from this trial suggested that the two treatments were about the same. We have outlined some of the more serious concerns regarding basic aspects of clinical trial design and conduct which could have perhaps been rectified by better reporting following the CONSORT statement fully (Moher et al., 2010). We look forward to the authors’ reply so that the study can be used effectively in future meta-analyses of topical treatments for AD (Chu et al., 2023).
